# Non-fasting changes of Hs-CRP level in Chinese patients with coronary heart disease after a daily meal

**DOI:** 10.1038/s41598-022-20645-2

**Published:** 2022-11-01

**Authors:** Qiu-Zhen Lin, Xue-Yan Zang, Yan Fu, Xingyu Wen, Qi-Ming Liu, Ling Liu

**Affiliations:** 1 Department of Cardiovascular Medicine, The Second Xiangya Hospital, Central South University, #139 Middle Renmin Road, Changsha, Hunan 410011 People’s Republic of China; 2 Modern Cardiovascular Disease Clinical Technology Research Center of Hunan Province, Changsha, People’s Republic of China; 3 Cardiovascular Disease Research Center of Hunan Province, Changsha, People’s Republic of China; 4 Research Institute of Blood Lipid and Atherosclerosis, Central South University, Changsha, Hunan 410011 People’s Republic of China; 5 Xiangya School of Medicine, Central South University, Changsha, Hunan People’s Republic of China

**Keywords:** Biomarkers, Medical research

## Abstract

High-sensitivity C-reactive protein (hs-CRP) is a key inflammatory factor in atherosclerotic cardiovascular diseases. In Chinese patients with coronary heart disease (CHD), the changes in hs-CRP levels after a daily meal and the effect of statins on those were never explored. A total of 300 inpatients with CHD were included in this study. Hs-CRP levels were measured in the fasting and non-fasting states at 2 h and 4 h after a daily breakfast. All inpatients were divided into two groups according to fasting hs-CRP ≤ 3 mg/L or not. Group with fasting hs-CRP ≤ 3 mg/L had a significantly higher percentage of patients with statins using ≥ 1 month (m) before admission than that with fasting hs-CRP > 3 mg/L (51.4% vs. 23.9%, *P* < 0.05). Hs-CRP levels increased significantly in the non-fasting state in two groups (*P* < 0.05). About 32% of patients with non-fasting hs-CRP > 3 mg/L came from those with fasting hs-CRP ≤ 3 mg/L. In conclusion, hs-CRP levels increased significantly in CHD patients after a daily meal. It suggested that the non-fasting hs-CRP level could be a better parameter to evaluate the inflammation state of CHD patients rather than fasting hs-CRP level.

## Introduction

High-sensitivity C-reactive protein (hs-CRP) is a well-known inflammatory factor, which is strongly associated with atherosclerotic cardiovascular diseases^1–3^. A large-sample study in Chinese ischemic stroke population showed that the higher hs-CRP level, the higher the risk of all-cause death would be^4^. Moreover, elevated hs-CRP level is also associated with the increased risk of major adverse cardiac events in patients after percutaneous coronary intervention^5^. Hs-CRP level > 3 mg/L is regarded as high hs-CRP level and predicts elevated risk for the first cardiovascular events^6^. Thus, hs-CRP level is an important predictor of cardiovascular events in primary or secondary prevention.

Serum hs-CRP level can be affected by a lot of factors, such as sex, age, waist circumference and systolic blood pressure^7,8^. In addition, food intake also exerts an important role in hs-CRP level. We previously observed that the non-fasting hs-CRP levels significantly increased at 4 h after a high-fat meal (800 cal, 50 g fat) only in non-coronary heart disease (CHD) patients at high risk for cardiovascular events, but not in healthy controls^9^. It suggested that the different populations could have different inflammation responses after a high-fat meal. For most of the day, humans are in the postprandial (i.e., non-fasting) state due to the eating habits, i.e., most people eat food three times a day. Until now, there was no study to explore the changes in hs-CRP levels after a daily meal in Chinese patients with CHD.

Statins using can reduce cardiovascular risk in both primary and secondary prevention through controlling cholesterol levels^10,11^. It was found that statins can reduce the cardiovascular events in asymptomatic individuals with elevated hs-CRP levels, even without hyperlipidemia^12,13^. We reported that fluvastatin (40 mg/day) effectively reduced the non-fasting hs-CRP levels after a high-fat meal in non-CHD patients at high risk for cardiovascular events^9^. However, the effect of statins using on the non-fasting hs-CRP levels after a daily meal instead of a high-fat meal in CHD patients is unclear.

In the present study, we aimed to learn about the fasting and non-fasting hs-CRP levels after a daily meal in hospitalized patients with CHD according to their eating habits, and to analysis the effect of long-term statins therapy on postprandial CRP levels in patients with different fasting CRP levels.

## Results

### Clinical characteristics and fasting biochemical examinations in two groups

There was no significant difference in age, sex, body mass index (BMI), heart rate, the percentages of smoking, hypertension, acute coronary syndrome or diabetes between two groups. CHD patients with fasting hs-CRP ≤ 3 mg/L had a higher percentage of cases with long-term [i.e., ≥ 1 month (m)] statins using than those with hs-CRP > 3 mg/L (51.4% vs. 23.9%, *P* < 0.05), while they had a lower percentage of cases with short-term (i.e., < 1 m) and without statins using than those with hs-CRP > 3 mg/L (48.6% vs. 76.1%, *P* < 0.05) (Table 1). Of all patients, 201 patients (67%) had taken statins before admission, while others (33%) did not take statins before admission.

**Table 1:** Comparisons of baseline characteristics between two groups.

	Fasting hs-CRP (mg/L)		*P*-value
	≤ 3 (n = 212)	> 3 (n = 88)	
Age, years	60.1 ± 10.0	61.3 ± 9.9	0.183
Male, n (%)	154 (72.6)	65 (73.9)	0.828
BMI, kg/m^2^	25.08 ± 3.36	25.01 ± 3.35	0.857
Smoking, n (%)	106 (50.0)	45 (51.1)	0.858
Hypertension, n (%)	153 (72.2)	68 (77.3)	0.361
ACS, n (%)	120 (56.6)	49 (55.7)	0.898
Heart rate, bpm	75.45 ± 14.68	78.5 ± 15.00	0.412
Diabetes, n (%)	60 (28.3)	22 (25.0)	0.559
**Statins using, n (%)**			
≥ 1 m	109 (51.4)	21 (23.9)	0.000
Without and < 1 m	103 (48.6)	67 (76.1)	0.000
TG, mmol/L	1.50 (1.07, 2.25)	1.40 (1.09, 1.99)	0.454
TC, mmol/L	3.85 ± 1.00	4.04 ± 0.97	0.096
LDL-C, mmol/L	2.34 ± 0.89	2.55 ± 0.84	0.020
HDL-C, mmol/L	1.02 ± 0.24	1.01 ± 0.23	0.939
hs-CRP, mg/L	1.05 (0.53, 1.88)	5.76 (4.25, 5.75)	0.000

The levels of blood lipids and hs-CRP were measured in the fasting state. Age, body mass index (BMI), heart rate, TC, LDL-C, HDL-C were expressed as mean ± standard deviation (M ± SD). TG and hs-CRP were expressed as median with 25th and 75th percentiles. Others were expressed as numbers and percentages.

There was no significant difference in fasting levels of triglyceride (TG), total cholesterol (TC) and high-density lipoprotein cholesterol (HDL-C) between two groups. The patients with fasting hs-CRP ≤ 3 mg/L had a significantly lower fasting level of low-density lipoprotein cholesterol (LDL-C) than those with hs-CRP > 3 mg/L (*P* < 0.05), which could be related to a higher percentage of long-term statins using (Table 1).

### Non-fasting changes in levels of blood lipids in two groups

**Figure 1:**
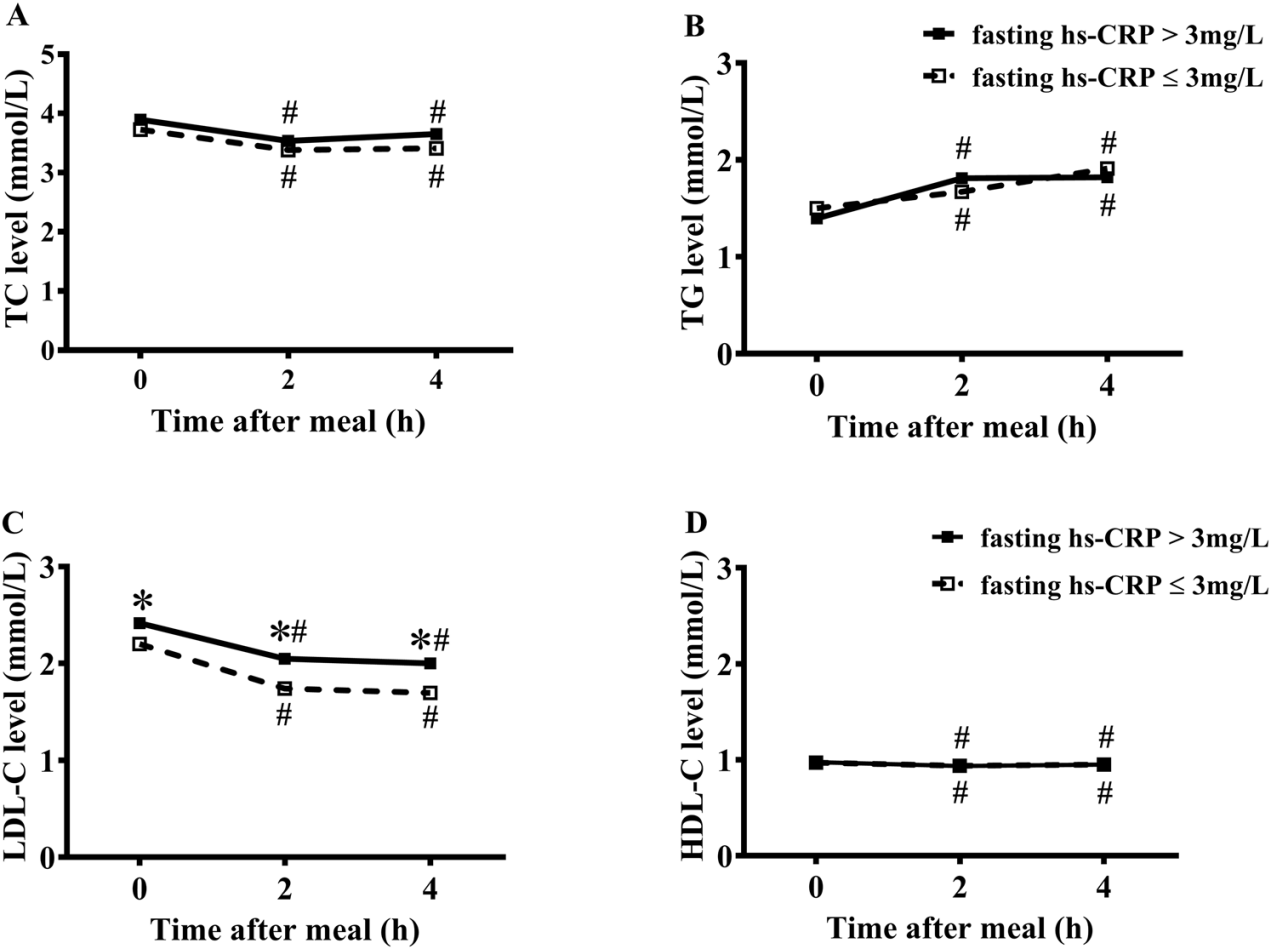
Comparisons of changes in levels of blood lipids after a daily meal between two groups. (**A**–**D**) The changed levels of TC (**A**), TG (**B**), LDL-C (**C**) and HDL-C (**D**) in two groups (i.e., CHD patients with fasting hs-CRP level ≤ 3 mg/L or not). *P < 0.05 when compared with fasting hs-CRP ≤ 3 mg/L group at the same time point, ^#^*P* < 0.05 when compared with the fasting concentration in the same group.

Levels of TC, HDL-C and LDL-C significantly decreased while level of TG significantly increased after a daily meal in two groups (*P* < 0.05). The changes in HDL-C levels after a daily meal were relatively slight in two groups (Fig. 1).

The patients with fasting hs-CRP ≤ 3 mg/L had significantly lower levels of LDL-C after a daily meal than those with hs-CRP > 3 mg/L (*P* < 0.05). However, there was no significant difference in the non-fasting levels of TC, TG and HDL-C between two groups (Fig. 1).

### Non-fasting changes in levels of Hs-CRP in two groups

**Figure 2:**
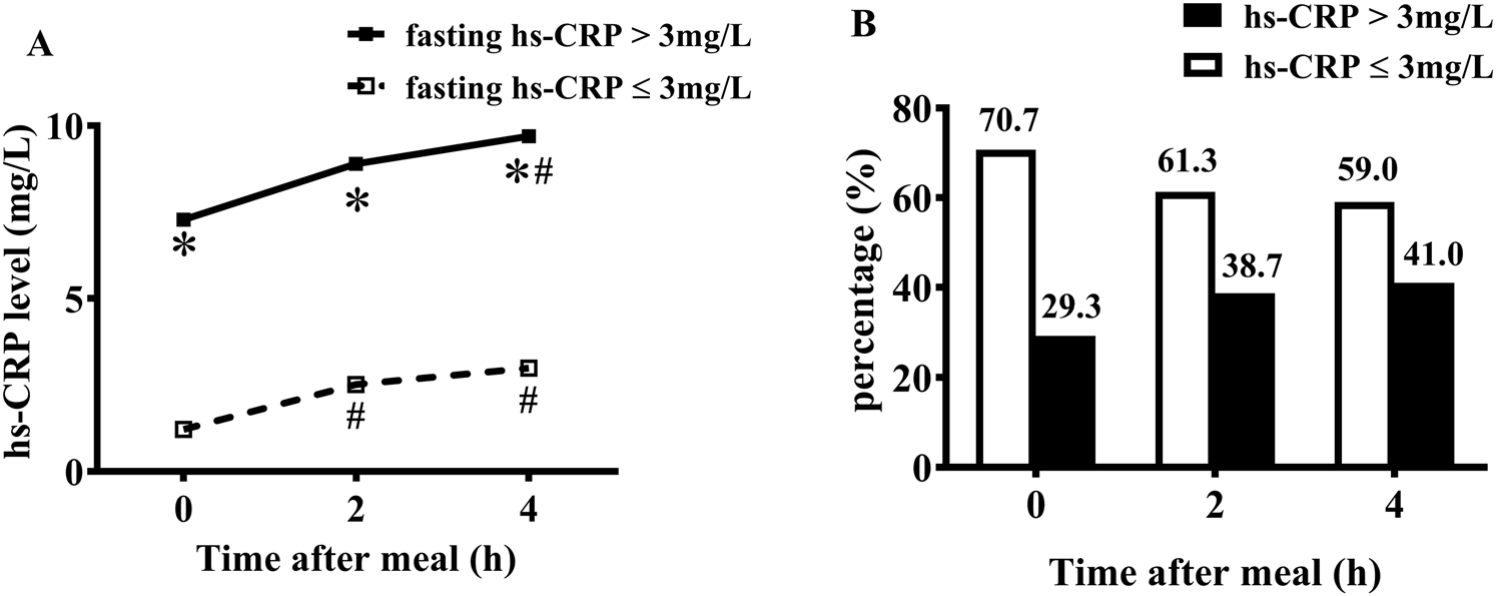
Comparisons of changes in hs-CRP levels after a daily meal between two groups. (**A**) Changes of postprandial hs-CRP level in two groups (i.e., CHD patients with fasting hs-CRP level ≤ 3 mg/L or not). (**B**) Comparisons of the percentages of patients with hs-CRP ≤ 3 mg/L and > 3 mg/L at different time points. *P < 0.05 when compared with fasting hs-CRP ≤ 3 mg/L group at the same time point, # P < 0.05 when compared with the fasting level within each group.

After a daily meal, hs-CRP level increased when compared with the fasting hs-CRP level in each group. However, it significantly increased at 4 h in the patients with fasting hs-CRP > 3 mg/L (*P* < 0.05) while at 2 h and 4 h in the patients with fasting hs-CRP ≤ 3 mg/L (both *P* < 0.05). The patients with fasting hs-CRP level > 3 mg/L had significantly higher levels of hs-CRP after a daily meal than those with fasting hs-CRP level ≤ 3 mg/L (*P* < 0.05) (Fig. 2A).

In either fasting or non-fasting state, the percentage of patients with hs-CRP level ≤ 3 mg/L was higher than those with hs-CRP level > 3 mg/L. Interestingly, the percentage of patients with hs-CRP level ≤ 3 mg/L decreased from fasting 70.7% to around 60% after a daily meal. However, the percentage of patients with hs-CRP level > 3 mg/L increased from fasting 29.3% to about 40% after a daily meal (Fig. 2B).

### Source analysis of patients with different Hs-CRP levels in the non-fasting state

Around 95% of the patients with non-fasting hs-CRP ≤ 3 mg/L at 2 h and 4 h derived from those with fasting hs-CRP ≤ 3 mg/L. Only about 5% of the patients with non-fasting hs-CRP ≤ 3 mg/L at 2 h and 4 h came from those with fasting hs-CRP > 3 mg/L.

Around 68% of the patients with non-fasting hs-CRP > 3 mg/L at 2 h and 4 h derived from those with fasting hs-CRP > 3 mg/L. About 32% of the patients with non-fasting hs-CRP > 3 mg/L at 2 h and 4 h came from those with fasting hs-CRP ≤ 3 mg/L (Table 2).

**Table 2:** Source analysis of patients with different hs-CRP levels in the non-fasting state.

Time	Fasting hs-CRP level (mg/L)	Percentage of subjects with different non-fasting hs-CRP level	
		≤ 3 mg/L (%)	> 3 mg/L (%)
2 h	≤ 3	94.4	31.2
	> 3	5.6	68.8
4 h	≤ 3	95.3	32.6
	> 3	4.7	67.5

Data were the percentage of CHD patients with non-fasting hs-CRP levels ≤ 3 mg/L or not after a daily meal deriving from those with different levels of fasting hs-CRP.

### Effect of statins using on non-fasting changes in Hs-CRP levels

**Figure 3:**
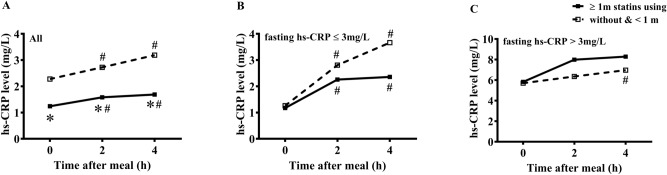
Comparisons of the changes in hs-CRP levels after a daily meal between CHD patients with different terms of statins using. (**A**) Comparisons of the changes in hs-CRP after a daily meal between patients with different terms of statins using in all subjects (i.e., ≥ 1 m statins using, n = 130 vs. without and < 1 m statins using, n = 170). (**B**) Comparisons of the changes in hs-CRP after a daily meal between patients with different terms of statins using in those with fasting hs-CRP level ≤ 3 mg/L (i.e., ≥ 1 m statins using, n = 109 vs. without and < 1 m statins using, n = 103). (**C**) Comparisons of the changes in hs-CRP after a daily meal between patients with different terms of statins using in those with fasting hs-CRP level > 3 mg/L (i.e., ≥ 1 m statins using, n = 21 vs. without and < 1 m statins using, n = 67). * *P* < 0.05 when compared with CHD patietns without or with statins using < 1 m at the same time point. ^#^*P* < 0.05 when compared with the fasting level within each group.

According to the condition of statins using, all patients were divided into two subgroups (i.e., ≥ 1 m statins using, n = 130 vs. without and < 1 m statins using, n = 170). Fasting level of hs-CRP in the subgroup with ≥ 1 m statins using was significantly lower than that in another subgroup. After a daily meal, hs-CRP levels at 2 h and 4 h significantly increased when compared with the fasting hs-CRP level in each subgroup (*P* < 0.05). Moreover, the subgroup with ≥ 1 m statins using had significantly lower hs-CRP levels at 2 h and 4 h than another subgroup (*P* < 0.05) (Fig. 3A).

The patients with fasting hs-CRP level ≤ 3 mg/L were divided into two subgroups (i.e., ≥ 1 m statins using, n = 109 vs. without and < 1 m statins using, n = 103) according to the condition of statins using. There was no significant difference in the fasting hs-CRP level between two subgroups. After a daily meal, hs-CRP levels at 2 h and 4 h significantly increased when compared with the fasting hs-CRP level in each subgroup (*P* < 0.05). The subgroup with ≥ 1 m statins using showed lower hs-CRP levels at 2 h and 4 h than another subgroup, however, the difference did not reach statistical significance (Fig. 3B).

The patients with fasting hs-CRP level > 3 mg/L were also divided into two subgroups (i.e., ≥ 1 m statins using, n = 21 vs. without and < 1 m statins using, n = 67) according to the condition of statins using. Level of hs-CRP increased after a daily meal in each subgroup, while the difference only reached statistical significance at 4 h in the subgroup without and with < 1 m statins using (*P* < 0.05). There was no significant difference in the fasting and non-fasting hs-CRP levels between two subgroups (Fig. 3C).

**Figure 4:**
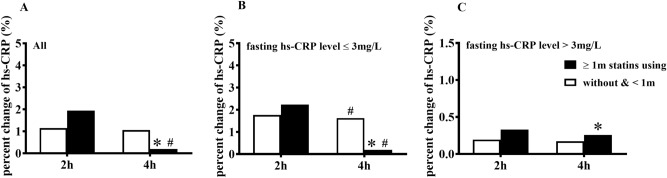
Comparisons of the percent changes of hs-CRP level between CHD patients with different terms of statins using. (**A**) Comparisons of the percent change of hs-CRP level at 2 h and 4 h after a daily meal between patients with different terms of statins using in all subjects (i.e., ≥ 1 m statins using, n = 130 vs. without and < 1 m statins using, n = 170). The percent change of hs-CRP level at 2 h was calculated by the formula: 100% × (hs-CRP_2h_-hs-CRP_0h_)/hs-CRP_0h_, the percent change of hs-CRP level at 4 h was calculated by the formula: 100% × (hs-CRP_4h_-hs-CRP_2h_)/hs-CRP_2h_. (**B**) Comparisons of the percent change of hs-CRP level at 2 h and 4 h after a daily meal between patients with different terms of statins using among those with fasting hs-CRP level ≤ 3 mg/L (i.e., ≥ 1 m statins using, n = 109 vs. without and < 1 m statins using, n = 103). (**C**) Comparisons of the percent change of hs-CRP level at 2 h and 4 h after a daily meal between patients with different terms of statins using among those with fasting hs-CRP level > 3 mg/L (i.e., ≥ 1 m statins using, n = 21 vs. without and < 1 m statins using, n = 67). *P < 0.05 when compared with those without and < 1 m statins using at the same time point. ^#^*P* < 0.05 when compared with the percent change of hs-CRP at 2 h after daily meal within each subgroup.

Either in all patients or within each group, there was no significant difference in the percent change of hs-CRP level at 2 h between two subgroups. Interestingly, the percent change of hs-CRP level at 4 h in the subgroup with ≥ 1 m statins using was not only significantly lower than that at 2 h within the same subgroup but also significantly lower than that at 4 h in another subgroup either in all patients or in the group with fasting hs-CRP level ≤ 3 mg/L (*P* < 0.05) (Fig. 4A, B). However, there was no similar phenomenon within those with fasting hs-CRP level > 3 mg/L (Fig. 4C).

Taking all patients as a whole (n = 300), correlation analysis showed that there was no significant relationship between the non-fasting change in hs-CRP level and that in either TG or LDL-C level at 2 h and 4 h (data were not showed), although both hs-CRP and TG levels increased after a daily meal.

## Discussion

Elevated hs-CRP level is an important factor predicting the increased CHD risk. It was found that hs-CRP level after a high-fat meal significantly increased in patients with high cardiovascular risk but not in healthy subjects^14^. The present study showed that CHD patients even after a daily breakfast could also cause non-fasting increase in hs-CRP level, regardless of the conditions of statins using and fasting hs-CRP level. It suggested that the non-fasting increase in hs-CRP level could be related not only to the content of meal^15^, but also to the underlying diseases and the associated cardiovascular risk factors of the individuals. More importantly, more than one-third of CHD patients with non-fasting high hs-CRP (i.e., > 3 mg/L) came from those with relatively low hs-CRP levels (i.e., ≤ 3 mg/L) in the fasting state. It supported that the non-fasting state is the key period of atherogenesis. And it suggested that the non-fasting hs-CRP level could be a better parameter to evaluate the inflammation state of CHD patients rather than fasting hs-CRP level.

Hs-CRP assay is a kind of hypersensitive detection technology used in the clinical laboratories, which can accurately detect low concentration CRP and improve the sensitivity and accuracy of the test. CRP is a nonspecific inflammatory marker secondary to the stimulation of interleukins (IL), including IL-1, IL-6 and IL-17, and the activation of the nuclear factor kappa B plays an important role in the regulation of CRP production^16,17^. CRP can be secreted by many cells, such as artery smooth muscle cells, aortic endothelial cells, inflammatory cells, pyramidal neurons, renal cortical tubular epithelial cells and so on, but it mainly comes from hepatocytes in vivo^16,18–20^. Liver connects with gastrointestinal tract directly via portal vein, and is sensitive to food intake. The digestive tract will transport large amounts of lipids to the liver through the portal vein when western food rich in sugar and fat is digested^21^. Under the stimulation of hyperlipidemia, the anti-inflammatory state of the liver will be transformed into an inflammatory state. No matter what the level of the fasting hs-CRP was, the two groups of CHD patients showed significant and similar increase in TG level after a daily meal. However, we did not find the close relationship between the non-fasting change in hs-CRP and that of TG in the present study, although high level of glucose or free fatty acid could play a potential role in the CRP production^22,23^. In a word, the non-fasting increase in hs-CRP level should not be ignored in CHD patients even after a daily meal.

In addition, diet-induced systemic inflammation accelerates IL production and release in other cells, which might also promote the production of CRP in the non-fasting state. It has been reported that IL-1β increased significantly in metabolic syndrome patients with central obesity after a high-fat meal, which was accompanied by prominently postprandial hypertriglyceridemia which represents the increased number of triglyceride-rich lipoproteins and their remnants^24^. It was demonstrated that chylomicron remnants promoted the expression of IL-1β in monocytes^25^. Moreover, we found that postprandial triglyceride-rich lipoproteins promoted premature aging of mouse subcutaneous adipose derived mesenchymal stem cells through oxidative stress mechanism, accompanying by the upregulation of IL-1α, IL-6 and other inflammatory factors^26^. As a key inflammatory factor in the upstream of inflammatory signaling pathway, IL-1β up-regulation or increased release can trigger a cascade of downstream inflammatory factors, including CRP^27,28^.

In the present study, there was no significant relationship between hs-CRP level and TG or LDL-C level, which may be due to the influence of lipid-lowering drugs. Because a considerable number of enrolled patients had taken statins before admission, which could have an impact on the level of hs-CRP. It was reported that statins play an anti-inflammatory role in hepatocytes via limiting pro-inflammatory genes expression, such as limiting nuclear accumulation and DNA binding of the nuclear factor kappa B^29^. Additionally, clinical trials also showed the anti-inflammatory role of statins^13,30,31^. It was consistent with the finding that the patients with statins using ≥ 1 m had significantly lower fasting and non-fasting hs-CRP levels than others in the present study.

However, statins using ≥ 1 m was not able to completely antagonize the rise of hs-CRP level caused by a daily breakfast, especially in those with fasting hs-CRP level > 3 mg/L. There was no deliberate diet control or standard meal in this study. A daily breakfast could be a high-fat meal or not for the different individuals. Dietary habits could be an important factor that affects blood lipids and the efficacy of lipid-lowering drugs even in the non-fasting state. The Centers for Disease Control and the American Heart Association had defined that fasting hs-CRP level ≤ 3 mg/L indicated lower or average cardiovascular risk while hs-CRP level > 3 mg/L indicated higher cardiovascular risk in 2003^32^. Under the influence of food intake, the non-fasting hs-CRP elevated to a higher level of hs-CRP (> 3 mg/L) in approximately 30% patients who had a lower level of fasing hs-CRP (≤ 3 mg/L). It indicated that a part of CHD patients with lower or average cardiovascular risk turned into those with higher cardiovascular risk in the non-fasting state. Considering that humans are in the non-fasting state in the most of a day, thus the detection of fasting hs-CRP level is not enough to reflect the real change in hs-CRP level throughout a day. It was confirmed that intermittent fasting was responsible to reducing risk factors of cardiovascular disease, such as fat mass, LDL-C and TG level^33,34^. However, it was still uncertain whether it will influence the cardiovascular risk deriving from hs-CRP elevation in the non-fasting state.

Elevated hs-CRP level could act as both inducer and indicator of CHD. It was reported that patients with CHD showed high hs-CRP level despite LDL-C < 70 mg/dL and consequently benefited from anti-inflammatory therapy^35^. The Controlled Rosuvastatin Multinational Trial also found that a long term rosuvastatin using could reduce fasting hs-CRP level and influence the cardiovascular events independent from LDL-C reduction^36^. However, the Heart Protection Study compared the vascular protective effects of simvastatin among 6 groups of patients with acute coronary syndrome and different fasting CRP levels, and found that simvastatin resulted in a significant reduction of vascular events (including coronary death, myocardial infarction, stroke, and revascularisation), but it did not modify the vascular benefits among groups with different baseline CRP levels^37^. However, the Canakinumab Anti-inflammatory Thrombosis Outcome Study (CANTOS) proved that reducing hs-CRP level through the IL-1β and downstream IL-6-receptor signaling pathway by canakinumab (a monoclonal antibody targeting IL-1β) without reducing LDL-C level significantly lowered the incidence of recurrent cardiovascular events^38^. Interestingly, the Cardiovascular Inflammation Reduction Trail (CIRT) using Methotrexate, a kind of drug had been successfully used at low doses to treat autoimmune diseases through blocking amido-imidazole-carbox-amido-ribonucleotide transformylase, to inhibit inflammation showed a completely contrary result compared with CANTOS^39,40^. Among patients with stable atherosclerosis, low-dose methotrexate did not reduce levels of interleukin-1β, interleukin-6, or CRP and did not result in fewer cardiovascular events than placebo. A possible explanation is that the baseline CRP level in CANTOS (4.2 mg/L) is significantly higher than in CIRT (1.6 mg/L), indicating that only patients with high baseline CRP level can benefit from additional anti-inflammatory therapy. Besides, the different anti-inflammatory drugs between above two studies could be another important factor inducing different results.

It seemed that long term statins treatment before admission played a certain role in lowering the non-fasting hs-CRP level. We found that statins using ≥ 1 m before admission could improve the fasting and non-fasting inflammatory response. However, the elevated hs-CRP level after a daily meal was not able to be antagonized in the patients with fasting hs-CRP level > 3 mg/L, although long term statins using was able to slow down the rise of hs-CRP level after a daily meal in those with fasting hs-CRP level ≤ 3 mg/L. It indicated that the statins using could be not enough to control the non-fasting inflammation in CHD patients with higher hs-CRP levels, and an additional anti-inflammatory therapy could be necessary for them.

## Conclusions

In conclusion, Hs-CRP levels increased significantly in CHD patients after a daily meal. About 32% of patients with non-fasting hs-CRP > 3 mg/L at 2 h and 4 h came from those with fasting hs-CRP ≤ 3 mg/L. It suggested that the non-fasting hs-CRP level could be a better parameter to evaluate the inflammation state of CHD patients rather than fasting hs-CRP level.

## Limitations

There were several limitations in this study. First, the investigation on the effect of statins using on hs-CRP level was a cross-sectional one rather than a prospective one. Second, the sample size in this study was relatively small, especially that of patients with higher hs-CRP level, and we will explore a larger, prospective and multicenter research in the future. Third, the breakfast contents of subjects were not recorded and analyzed in detail.

## Methods

### Subjects

A total of 300 inpatients at the Department of Cardiovascular Medicine in the Second Xiangya Hospital of Central South University were recruited in this study from August 2015 to October 2019. The cohort included 300 patients with CHD. CHD was defined as a history of myocardial infarction (MI) and/or angiographically proven coronary atherosclerosis in patients with angina pectoris (AP)^41^. Contemporaneous subjects who had no clinical history and manifestation of CHD were excluded. According to fasting level of hs-CRP > 3 mg/L or not, patients with CHD were divided into two groups, i.e., group with fasting hs-CRP > 3 mg/L or not.

The study was approved by the Ethics Committee of the Second Xiangya Hospital of Central South University and conformed to the 1975 Declaration of Helsinki. Informed consent was gained from all subjects.

### Laboratory examinations

All enrolled subjects took the breakfast according to their daily diet habits at 7 am to 8 am after an overnight fast at least 8 h, and their breakfasts were purchased from the hospital cafeterias or brought home. It included a variety of common Chinese breakfasts, such as noodles, porridge, dumplings, steamed stuffed buns, eggs and fried dough sticks. Venous blood samples were collected in all persons in the fasting state and at 2 h, 4 h after a daily breakfast. Serum levels of TC and TG were measured by automated enzymatic assays, and those of LDL-C and HDL-C were measured by a commercially available direct method (Wako, Japan), i.e., selective protection method and antibody blocking method, respectively, on a HITACHI 7170A analyzer (Instrument Hitachi Ltd., Tokyo, Japan) by a laboratory technician who had no idea of this study^42^. Hs-CRP was analyzed from samples with a latex turbidimetric immunoassay (Medicalsystem, Ningbo, China). The analytical detection limit for this method is 0.5 mg/L.

### Statistical analysis

Statistical analysis was performed on SPSS 24.0. Data drawing was completed by Graphpad Prism 7.0 software. Quantitative variables were expressed as mean ± standard deviation, and qualitative variables were expressed as numbers and percentages. The percent change of hs-CRP level at 2 h was calculated in accordance with the formula: 100% × (hs-CRP_2h_-hs-CRP_0h_)/hs-CRP_0h_, the percent change of hs-CRP level at 4 h was calculated in accordance with the formula: 100% × (hs-CRP_4h_-hs-CRP_2h_)/hs-CRP_2h_. Fasting TG and hs-CRP levels not conforming to a normal distribution were log transformed for comparison and presented as median with 25th and 75th percentiles. Differences between the intra- and intergroup means were analysed by student’s *t*-test. The statistical differences of non-normal distribution data were tested by Mann–Whitney nonparametric test. Categorical variables were compared using chi-squared statistic tests. All *P* values were 2-tailed, and *P* < 0.05 was considered statistically significant.

### Ethics statement

All procedures performed in this study involving human participants were in accordance with the ethical standards of the institutional and/or national research committee and with the 1964 Helsinki declaration and its later amendments or comparable ethical standards. The data collection procedure was discussed and approved by the Ethics Committee of the Second Xiangya Hospital of Central South University.

## Data Availability

The datasets used and/or analysed during the current study available from the corresponding author on reasonable request.
